# Monitoring and Evaluation of Alcoholic Fermentation Processes Using a Chemocapacitor Sensor Array

**DOI:** 10.3390/s140916258

**Published:** 2014-09-02

**Authors:** Petros Oikonomou, Ioannis Raptis, Merope Sanopoulou

**Affiliations:** 1 Department of Microelectronics, Institute of Nanoscience and Nanotechnology (INN), NCSR ‘Demokritos’ Athens 15310, Greece; E-Mail: raptis@imel.demokritos.gr; 2 Department of Physical Chemistry, Institute of Nanoscience and Nanotechnology (INN), NCSR ‘Demokritos’ Athens 15310, Greece; E-Mail: sanopoul@chem.demokritos.gr

**Keywords:** alcoholic fermentation spoilage, gas sensing system, chemocapacitors, PCA

## Abstract

The alcoholic fermentation of *Savatiano* must variety was initiated under laboratory conditions and monitored daily with a gas sensor array without any pre-treatment steps. The sensor array consisted of eight interdigitated chemocapacitors (IDCs) coated with specific polymers. Two batches of fermented must were tested and also subjected daily to standard chemical analysis. The chemical composition of the two fermenting musts differed from day one of laboratory monitoring (due to different storage conditions of the musts) and due to a deliberate increase of the acetic acid content of one of the musts, during the course of the process, in an effort to spoil the fermenting medium. Sensor array responses to the headspace of the fermenting medium were compared with those obtained either for pure or contaminated samples with controlled concentrations of standard ethanol solutions of impurities. Results of data processing with Principal Component Analysis (PCA), demonstrate that this sensing system could discriminate between a normal and a potential spoiled grape must fermentation process, so this gas sensing system could be potentially applied during wine production as an auxiliary qualitative control instrument.

## 1. Introduction

The task of monitoring and evaluating the alcoholic fermentation of grape must to wine with techniques other than conventional oenological processes is challenging. Many efforts have been recorded in the field of chemical/biochemical sensing targeting at this specific application. Several of them have focused on sensor arrays based on different type of transducers supplemented with gas chromatography and mass spectrometry (GC/MS) techniques [1–3]. Until recently the GC/MS method has been considered the standard analytical tool for fermentation control, however the relatively high-cost and “off-line” nature of the analyses has tended to restrict its use in fermentation monitoring.

Some researchers use even more complementary complex sample pre-treatment steps such as headspace/solid phase microextraction (HS/SPME), purge and trap systems or liquid–liquid extraction systems or a combination of them [4–7]. These selective HS/SPME and purge and trap systems, equipped with very hydrophobic polymeric membranes, such as 2,6-diphenyleneoxide polymer resin with 30% graphite content and using thermal desorption techniques, provide the necessary sensitivity for the analysis of low-boiling point compounds e.g., acetaldehyde and ethyl acetate in fermentation samples. On the other hand, liquid-liquid extraction methods allow for the analysis of a great number of medium to high boiling point volatile compounds. The latter case may require solvent evaporation, which can be responsible for the loss or degradation of some compounds and/or the formation of others not present in the original wine.

It is necessary to mention that more than 800 different chemicals have been reported to be present in the volatile fraction of wines, but the number of odorants present in concentrations above the sensor detection threshold is at least one order of magnitude lower and the most of them require extremely complicated and expensive analytical methods for their detection. Besides, in many wines there are no key compounds that determine their aroma profile, which is due to the mixture of different odorants. Not all the volatile compounds have a considerable contribution in wine aroma. The role of each compound is known to be a function of concentration as well as sensory threshold (*i.e*., the minimum concentration that can be perceived by the human nose).

The most common method to address aromatic properties is the so-called sensorial panel test. This method, nevertheless, requires the training of human specialists. Certain disadvantages, such as the high cost and the difficulty in setting standards for an objective estimation, preclude a widespread application of this procedure.

Alternatively, olfactory assessment focuses on systems that are fast, non-destructive and objective, at a reasonably low cost, as compared to standard analytical methods. The use of arrays of gas sensors, also known as electronic noses (e-noses), with purposely-designed software for discrimination of signals, is increasingly applied. The sensing principle, in general, is based on the measurement of the variations of the gravimetric, optical, calorimetric or electrical properties of the active materials [8–16]. This approach seems to be useful for acquiring the information about certain characteristics of the measured object (fermenting grape must), rather than about its defined elements, since the sensor array consist of partially selective sensors. Each sensor of the array responds to a certain group of chemical compounds showing in most cases a broad and therefore overlapping response to the individual substances. This behavior allows sensing of complex aroma profiles. For each complex vapor environment the sensor array produces a unique response pattern, designated as a “fingerprint”. Each fingerprint contains the information for the specific vapor environment. In that case the implementation of adequate data processing techniques is required for unfolding that information. Several data processing techniques are reported. The use of mathematical algorithms like principal component analysis (PCA) [17–20], partial least square regression (PLS) [21] or artificial neural networks (ANN) [22] can remarkably enhance the data processing. Also, trained neural nets [23] are able to recognize measurement errors or obtain data from disturbed measurements.

In the present work we deal with the task of monitoring and evaluating the alcoholic fermentation of a typical Greek grape must variety, under laboratory conditions, with the use of a of a sensor array composed by eight interdigitated chemocapacitors (IDCs). For this particular application no pre-treatment steps or scientific equipment is required, since the interference signal of the high moisture levels of the fermenting must headspace is eliminated by the applied experimental protocol. Interfacing of the sensor array with the vapor environment is based on the dynamic headspace analysis technique. The latter allows the analysis of the volatile fraction without necessarily destroying it. The method involves purging the sample with an inert gas in much the same way as we breathe in the natural flavour of a product, and permits a correlation with sensory studies. Subsequently the data acquisition and processing of the sensor array responses is realized with PCA. It should be stressed that PCA does not provide any direct information on chemical composition of a mixture or concentrations of its compounds, but rather it represents both, indirectly. Complementarily an “off line” conventional laboratory chemical analysis of the fermented musts is performed in order to define volatile components present in elevated concentrations, and therefore the sensor array fingerprint. Those components are either indicators of the potential spoilage of the olfactory properties of the final product or responsible for stuck/sluggish fermentations.

## 2. Experimental Section

### 2.1. Fermentation Process

Two batches of a commonly used, in Greek winemaking tradition, Savatiano grape must variety, were used. Both batches were treated with a suitable quantity of K_2_S_2_O_5_ (approximately 150 mg/L sulfur dioxide) and then stored in a fridge for 1 month before use. The fermentation process was initiated by inoculating, at ambient temperature, with *Saccharomyces cerevisiae* yeast in a stainless steel fermenter. The two fermentation processes were realized under the same laboratory conditions, with 5 L of each must (denoted as Must 1 and Must 2) after the appropriate correction steps limiting the growth of spoilage microorganisms *i.e*., correction of must's initial total acidity expressed as tartaric acid (g/L) (must be in the range 6–8 g/L), adjustment of pH value (3.2–3.7), activation of the anhydrous yeast strains in 5% (w/w) sugar (aq) and inoculation of the just hydrated yeast strains in the must (optimal concentration 20 g/L) [24,25]. The fermentation temperature was controlled to 20 ± 1 °C.

Fermentation of Must 2 was intentionally disturbed by adding acetic acid (2 g/L) at Day 5 of the fermentation process. High levels of acetic acid are often associated with stuck or sluggish fermentations. Elevated acetic acid concentrations can inhibit cell growth, enhance ethanol toxicity and prevent the completion of fermentation [26]. Fermentation inhibition due to elevated concentrations of acetic acid is correlated with a decrease of the internal pH of the fermenting yeast [27,28]. While cell growth is in progress the presence of ethanol potentiates both the inhibition of fermentation and the internal acidification originated by acetic acid. On the contrary when the cell growth stage is completed (stationary phase) the ethanol production may still proceed even in the presence of acetic acid [29]. Therefore, acetic acid is potentially responsible for fermentation problems.

### 2.2. Chemical Analysis and Sensor Array Measurements

On a daily basis a 200 mL/50 mL sample of fermented must was ex**t**racted from the batch for chemical analysis and sensor array measurements, respectively. The protocol for the chemical analysis was based on conventional enological processes. Must was distilled and sugar, ethanol and ester content, the latter expressed as ethyl acetate (ppm), were measured by refractometry, density measurements and photometrically, respectively [25,30]. Titrimetry was used for estimating the total acidity (in the must) and volatile acidity (in the distillate after steam distillation). As stated in the Introduction section, the wine headspace must contains a wealth of compounds in minute quantities, however elevated quantities of ethyl acetate and acidity are indicators of possible spoilage of wine aroma and/sluggish fermentations.

**Figure 1.**
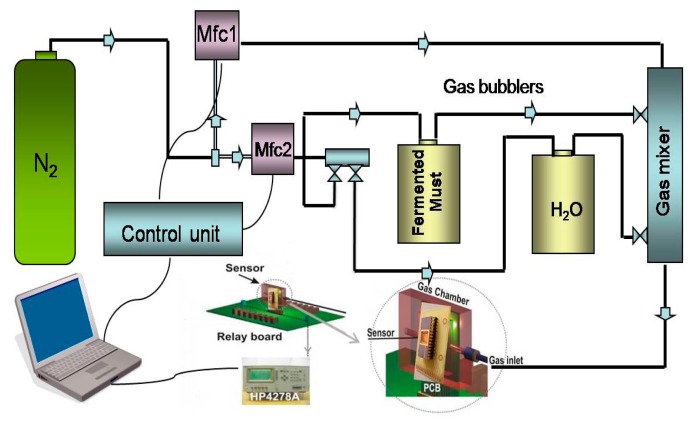
Experimental set up for the interface of the volatile headspace of the examined fermented medium with the sensor array.

The evaluation of the sensors' response to the fermenting must headspace was performed by the bubbling technique with the use of a computer LabView software controlled vapour delivery set up [31] (Figure 1).

In the gas-delivery unit, initially a dry nitrogen flux is split into a carrier and a dilution part with the help of two mass flow controllers (Mfc). The carrier is bubbled through the liquid analyte of interest and subsequently mixed with the dilution flow. The experimental protocol is designed as follows: initially the carrier part passes through the bubbler containing water and when a sensor equilibrium response is reached, it passes with the same flow rate through the bubbler containing a sample of the fermented must. The characterization set up as follows: the setup shown in Figure 1 is placed in a temperature controlled chamber and all the measurements were performed at the same temperature of the fermentation process, 20 ± 1 °C. The gas delivery unit (mass flow controllers (Mfc1 and Mfc2), valves, temperature) is controlled with the appropriate built-in LabView software.

The reference vapour was concentrated humid air, produced by bubbling pure water instead of the must. In this way the effect of the interference signal of water vapour, due to the very high water concentrations in the fermented must, upon exposure of the sensors to fermenting must is at least partially reduced. To further understand if these sensors can only perceive the evolution of ethanol during the fermentation a comparison with equilibrium values upon exposure to standard ethanol solutions is implemented.

### 2.3. Sensor Array Fabrication

**Figure 2.**
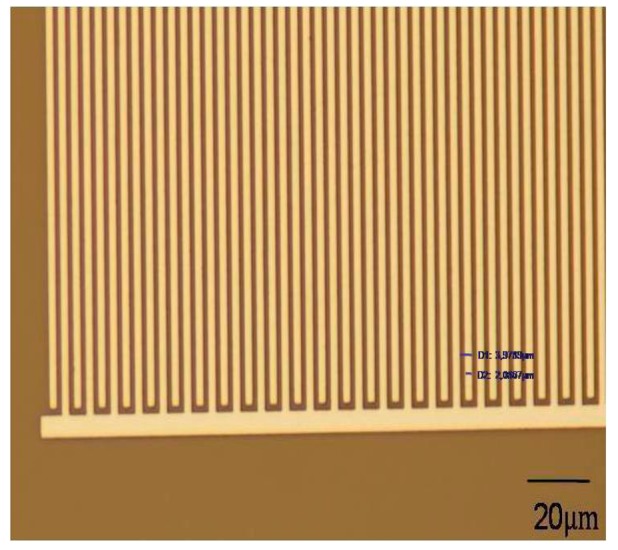
Optical micrograph of the IDE dimensions.

The chemocapacitor array is fabricated with standard microelectronic/micromachining processes allowing for the realization of interdigitated electrodes (IDEs) with critical dimensions of 2 μm (finger width (W) of electrodes equal to gap (G) between them) (Figure 2). Each chip has an area of 7 × 7 mm^2^ and consists of eight IDCs.

The selection of this particular planar layout for the IDEs is based on the results of a previous study where a simulation model, based on finite element analysis, for the prediction of the capacitance of different IDEs geometries was developed [32,33]. Each IDC has a 1 mm^2^ sensing area and the initial capacitance of the uncoated IDC is ∼6 pF. Around each IDC a well of a thick epoxy-based resist layer of ∼50 μm height is formed. The deposition of the sensing polymeric film was achieved by drop casting, within the epoxy well, of appropriate volumes of 10% (w/w) polymeric solutions to produce dry film thicknesses high enough to cover the electric field lines of the IDEs [height, h > 0.5λ = 2(W + G)]/2 = 4 μm]. This way controllable and repeatable deposition of the polymeric film, that acts as the sensing layer on the IDEs, without deterioration of the sensing response, is achieved. Since the thickness (h) of the polymeric layer is higher than half the periodicity (λ) of the electrodes, the capacitance changes upon exposure to vapor analytes is mainly produced by dielectric constant changes, which in turn are determined by the sorption capacity of the polymeric material to a particular analyte and by the relative values of the dielectric constant of polymer and analyte.

The following polymers were selected for casting: poly(dimethylsiloxane-co-diphenylsiloxane) dihydroxy terminated-PDMS-OH, poly(2-hydroxyethyl methacrylate)-PHEMA, poly(*n*-butyl methacrylate)-PBMA, poly(isobutyl methacrylate)-PiBMA, poly(ethyl methacrylate)-PEMA, poly(styrene)-PS, poly(ethylenimine)-PEI, and poly(vinylpyrrolidone)-PVP. The polymers were selected on the basis of previous studies of their sorptive capacity to water vapor, low molecular weight alcohols and volatile esters [34], as well as the sensitivity and partial selectivity of the corresponding sensors in pure VOCs, and humidity [35]. For example, sorption studies have shown that: (i) among the relatively hydrophobic methacrylic polymers, PiBMA was the most suitable for discriminating alcohols from water and (ii) PDMS-OH has a higher affinity for ethyl acetate as compared to ethanol [34]. In addition, IDC sensors based on PDMS-OH, PBMA and PEMA were found to be the less sensitive to humidity (sensitivity dC/Co/Cg < 7 ppm^−1^ in all three cases) [35]. For the drop casting procedure of the sensing polymeric layers, 10% (w/w) polymer solutions were prepared with the following solvents: methyl isobutyl ketone for PDMS-OH, ethyl lactate for PVP, methanol for PHEMA and PEI and propylene glycol monomethyl ether acetate for PBMA, PiBMA, PEMA and PS, respectively. All the polymers and solvents were obtained by Sigma Aldrich (Athens, Greece). After deposition of the polymeric films by drop casting, the sensor is adjusted in a dual in- line package and wire bonded. All measurements performed with a HP 4278A capacitance meter (Agilent Technologies, Santa Rosa, CA, USA) at 1 MHz.

### 2.4. Evaluation of the Fermentation Procedure

The equilibrium responses of the sensor array to daily collected samples of fermented must were processed with PCA. In particular the input data are the equilibrium responses of each sensor of the sensor array dC (pF) (dC is defined as the difference between the equilibrium responses to the vapours of the fermented must and to the vapour of pure water, respectively). The PCA statistical method that was applied includes the following steps: (i) calculation of the covariance matrix for all the input data. In our case variables are the equilibrium responses of each sensor and objects are the different samples (sample of fermenting Must 1 at Day 1, sample of fermenting Must 1 at Day 2,…, sample of fermenting Must 2 at Day 1,…); (ii) extraction of the eigenvectors and their corresponding eigenvalues; (iii) estimation of the representativeness of the eigenvectors; (iv) building of the feature matrix which includes the dominant vectors and (v) projection to the score matrix. A correlation between the sensor array responses obtained during fermentation progress and standard ethanol solutions is also performed. This way an overall view of the fermentation processes is obtained.

## 3. Results and Discussion

### 3.1. Chemical Analysis

**Figure 3.**
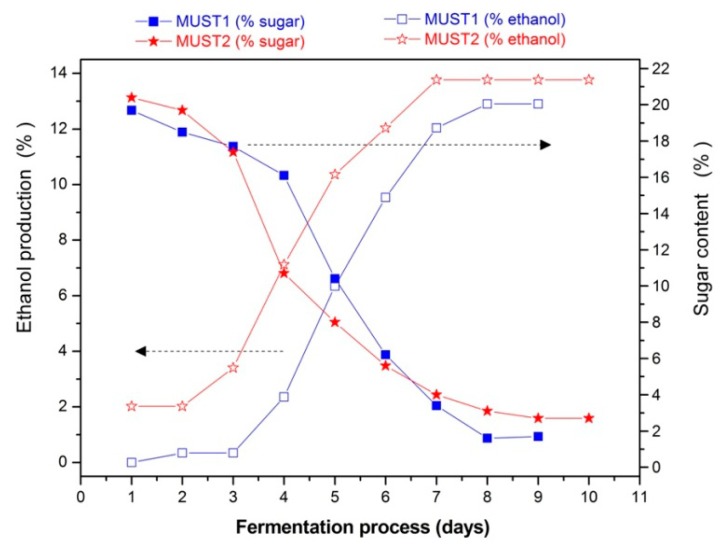
The evolution for the two different fermentation processes over time after daily chemical analysis in terms of sugar consumption and ethanol production.

**Figure 4.**
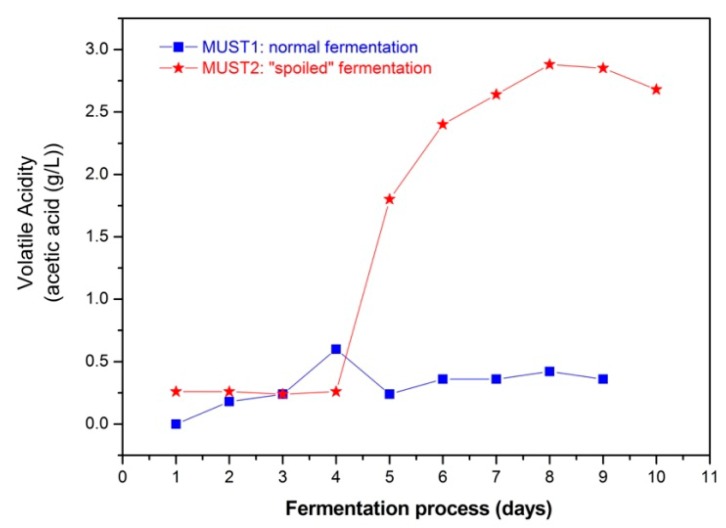
Volatile acidity propagation during the two fermentation processes. The results obtained by standard chemical analysis with steam distillation and titrimetry.

The evolution of the fermentation processes is determined by chemical analysis. Figure 3 shows the sugar consumption and the ethanol production during fermentation and Figure 4 the evolution of volatile acidity of the two fermenting musts. In the latter figure, the increase in volatile acidity from Day 5 of the process in Must 2, resulted from adding 2 g/L of acetic acid in the must. Finally, in Table 1 the initial and final volatile esters and total acidity are presented.

As illustrated in Figure 3, the two fermentations followed a sigmoidal function over time for both ethanol production and sugar consumption. This behavior is, in general, characteristic of a typical alcoholic grape must fermentation without defects. However, several differences between the two processes appear. In the first place, we note that Must 2 is characterized by initial higher ethanol content (Figure 3), indicating that on Day 1 of laboratory monitoring, the fermentation has already begun during the storage period. In line with this, is the corresponding initial higher amount of volatile esters (Table 1) as compared to Must 1.

The addition of excess acetic acid at Day 5 of Must 2 fermentation (Figure 4) did not appear to slow down the rate of ethanol production (Figure 3). This is probably due to the fact that Must 2 at Day 5 was already partially fermented, with an ethanol content of 10.36% (v/v) and cell growth was completed, while the toxic effect of acetic acid is expected to be more evident during the cell growth stage of glucose-grown populations of *Saccharomyces cerevisiae* [29,36]. Thus, according to chemical analysis, the fermentation process of Must 2 is characterized by higher initial ethanol and acetate content and elevated, purposely, higher acidity after 5th day of the fermentation process. These differences may also affect the overall composition of the must headspace during the process. The results presented in the next section aim at investigating the ability of the sensor array to monitor and distinguish between the evolution of the two different processes and provide a different PCA fingerprint for each fermentation.

### 3.2. Evaluation of Sensor Responses

**Figure 5.**
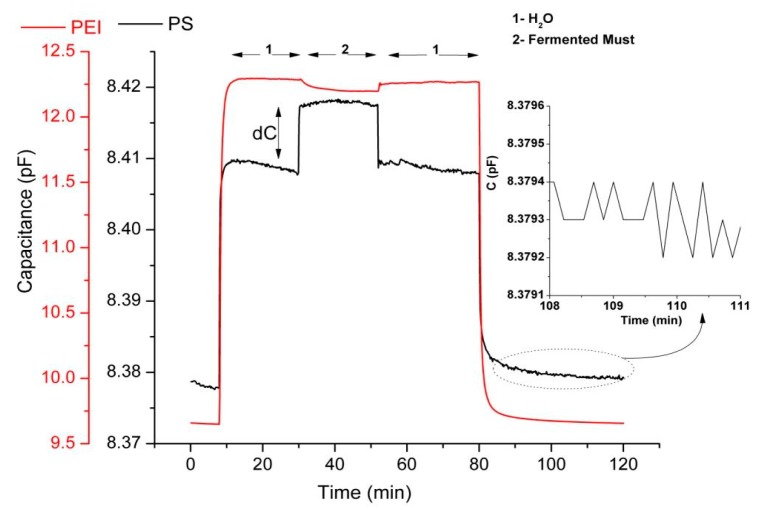
Typical example of dynamic responses of two sensors of the sensor array upon exposure to reference and fermented must. (Sample of fermented must: Must 1 fermentation process-Day 6). Also zoom in PS-coated sensor response is shown (insert graph).

The fermentation process is a “live” ongoing process, meaning that the composition is changing with time. Every 24 h a sample is taken for the capacitance measurement and for the chemical analysis. Therefore, only one capacitance measurement is performed every day. A characteristic example of the dynamic responses of two sensors of the sensor array according to the described experimental protocol is presented in Figure 5. The responses recorded (which were also the input data for principal component analysis) are the differential equilibrium responses between the plateau capacitance value upon exposure to vapours of the examined sample (standard aqueous ethanol solutions or samples of the fermented must) and the plateau capacitance value upon exposure to vapours of pure water. By using pure water headspace as reference analyte we excluded the interference signal in the sensor response due to the high humidity concentration in fermented must (∼80% (v/v) in must and ∼85% (v/v) in wine). As shown in Figure 5 (zoom in), the measurement noise is approximately 0.0003 pF for the PS-coated sensor. Therefore, responses above 0.001 pF, according to acceptable signal to noise ratio (S/N ≥ 3), are readable. The equilibrium responses of each sensor to the headspace of the fermenting musts were recorded daily.

**Figure 6.**
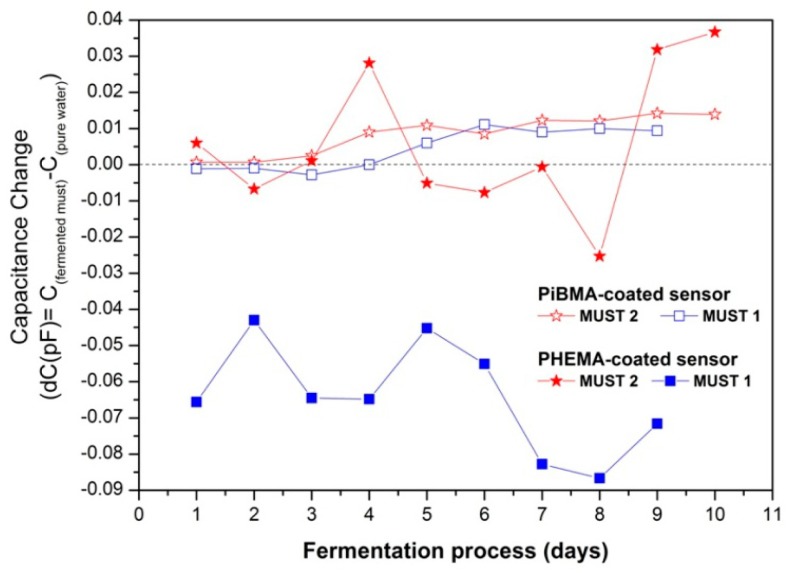
Differential equilibrium responses of the relative hydrophobic P(iBMA)-coated sensor and the relative hydrophilic PHEMA-coated sensor over fermentation duration.

In Figure 6 the equilibrium responses of different sensors of the array, showing distinctly different response behaviors during the fermentation process, are presented. In general the response of a sensor to a single analyte of a given vapor concentration is determined by two factors: the sorptive capacity of the sensing polymeric layer to this analyte, and the dielectric constant of the analyte [37]. Moreover, when monitoring the headspace of a multicomponent liquid mixture, the volatility of each component is affected by the presence of the other constituents of the mixture [38].

The PiBMA-coated sensor is inert to humidity, but relatively sensitive to sorption of alcohols and other produced volatile compounds [34]. Therefore its capacitive response was positive for both fermentation processes, although the alcohols have considerably lower dielectric constants as compared to the reference analyte, water (*i.e*., for ethyl alcohol ε = 24.6 and for H_2_O ε = 80.0). This behavior is characteristic of relatively hydrophobic polymer-coated sensors (*i.e*., PBMA, PiBMA, PS, PDMS-OH and PEMA).

On the contrary, for the PHEMA coated sensor, due to its pendant OH groups, enhanced hydrogen-bonding ability and polarity is expected. PHEMA polymeric films were tested upon exposure to vapours of pure analytes and correlation of the corresponding to hydrogen-bonding and polar interactions solubility parameters in conjunction with sorption ability has been performed. These studies showed higher and similar affinity between PHEMA and alcohol or water than ester content [34,39]. The latter, for the normal fermentation process resulted in a negative response to the headspace of the alcohol-rich fermenting must, possibly due to the lower volume fraction of water in the must, as compared to the reference and the lower dielectric constant of any sorbed organic compound as compared to water. Nevertheless for the case of Must 2 fermentation process, at the early stage the presence of higher concentrations of ethanol content in conjunction with the production of minimal concentrations of other low dielectric permittivity volatile compounds result in a diverse behavior. Since the headspaces of fermented musts are multi-component vapour environments where the volatility of each component is affected by the presence of the other constituents, even small concentration changes could affect the sensors response. Beyond Day 5, its response is also affected by the presence of high concentrations of acetic acid in the fermented medium. The same behavior was observed for all the relatively hydrophilic polymers (*i.e*., PHEMA, PVP, PEI).

For the evaluation of the experimental data, further information about each sensor response was obtained by exposing the sensing system to the headspace of standard aqueous solutions of ethyl alcohol. These solutions were either pure or contaminated with controlled concentrations of ethyl acetate and acetic acid, in an effort to simulate the environment of the fermenting must headspace.

In Table 2 the equilibrium responses of two representatives of the two sets of polymer-coated sensors, upon exposure to the headspace of several standard pure or contaminated ethanol solutions are illustrated. The capacitance measurement of the standard aqueous ethanol solution is repeated three times, resulting in a mean value with the corresponding standard deviation.

As before, the equilibrium response profile was similar for all relative hydrophobic and relative hydrophilic polymer-coated sensors, respectively. For both sets, a slight negative response is observed upon exposure to the headspace of pure acetic acid (aq) due to its lower dielectric constant (ε = 6.2) as compared to water. Strong negative responses were not observed, probably due to the formation of H-bonds between acetic acid and water and their similar volatility [40]. This negative response contributes to the overall response in an additive way, *i.e*., lowering the value of ethanol solutions response below that of pure standard ethanol solution. On the contrary ethyl acetate has a positive effect for both PBMA and PEI. As a result, the mean responses for pure 12% (w/w) ethanol solution and the corresponding one containing acetic acid and ethyl acetate—at similar proportions to that of the Must 2 fermentation process—are approximately the same (first and last column of Table 2).

**Figure 7.**
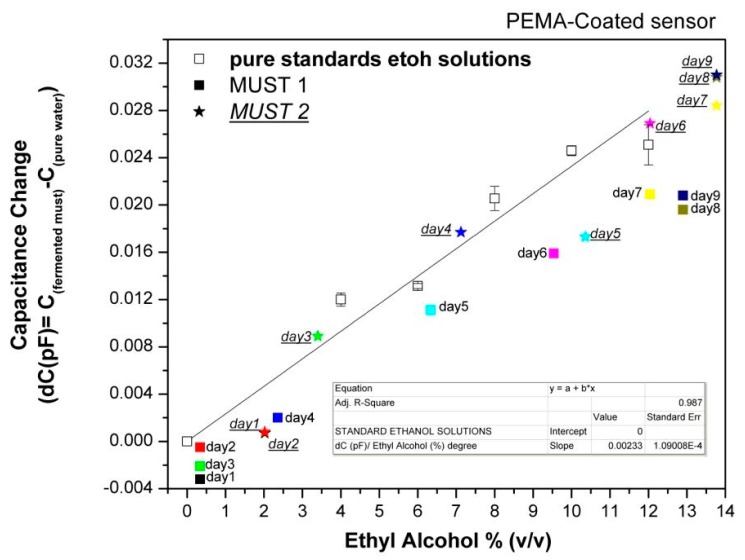
Equilibrium responses of a relative hydrophobic polymer-coated sensor upon exposure to the headspace of pure ethanol solutions in comparison with data of fermentations.

**Figure 8.**
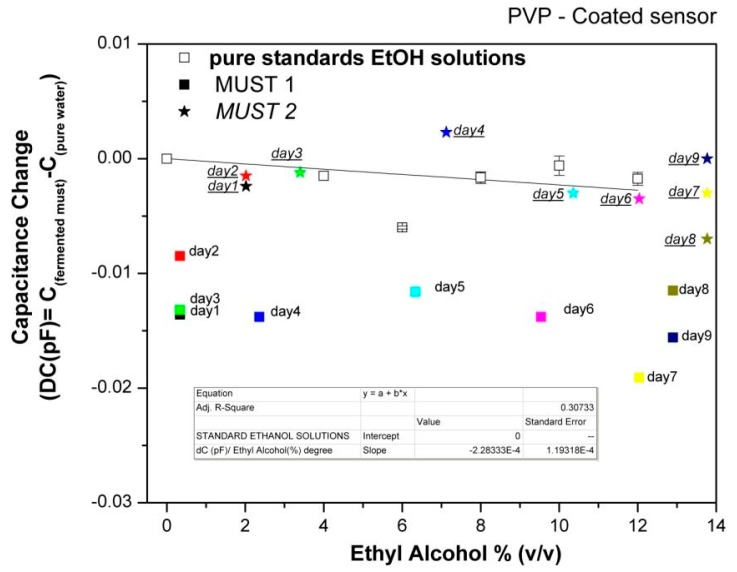
Equilibrium responses of a relative hydrophilic polymer-coated sensor upon exposure to the headspace of pure ethanol solutions in comparison with data of fermentations.

In order to further evaluate the array's responses to the fermenting musts, the capacitance data collected at each day of the fermentation process were plotted *vs*. the corresponding ethanol content of the musts, as deduced by chemical analysis, and compared with the sensor responses to pure ethanol solutions in the concentration range of 0%–12% (v/v). Characteristic examples are shown in Figures 7 and 8.

The data of Figures 7 and 8 permit several useful observations. For the PEMA-sensor it is obvious that positive responses occur with the initiation of ethanol production and the responses increase with increasing ethanol content. By fitting the responses to the headspace of standard ethanol solutions, a capacitance change of 0.0023 pF per degree of alcohol, is calculated. Thus, since the minimum readable response is 0.001 pF, the limit of detection in terms of alcohol degree is ∼0.5% (v/v). The differences between standard ethanol solutions and musts of the same ethanol concentration are attributed to the presence of other volatile compounds in the musts.

For the hydrophilic PVP polymer, the corresponding fitting reveals a relative insensitivity to standard ethanol solutions compared to headspace of pure reference analyte. The higher dielectric constant value of water as compared to that of ethanol and the rather dilute solutions studied (up to 12% (v/v)) affect the responses of pure alcohol solutions in such a way that they are not differentiated from those of the reference signal (pure water), *i.e*., the strong water signal masks that of ethanol, indicating a competitive role between sorption of ethanol and the reduction of the volume fraction of water.

Ethanol solutions generally show higher responses than Must 1 of the same ethanol concentration, indicating that other volatile compounds present in the must be contributing to their response. However, responses of Must 2 are differentiated from Must 1 and are close to those of pure ethanol solutions. As discussed above, Must 2 at Day 1 of laboratory monitoring has higher ethanol content as well as higher ethyl acetate content, indicating that the fermentation was already in progress. The addition of acetic acid at the 5th day of monitoring did not stop the alcohol production rate, but changed the chemical composition from the 5th day on. Thus, there is evidence of an overall different chemical composition of the two musts during the whole fermentation process, which results in different responses of the hydrophilic sensors. Accordingly, we may conclude that the hydrophilic sensors are not sensitive to the rate of alcohol production but to the overall complex composition of the must.

### 3.3. Evaluation of the Fermentation Procedure

**Figure 9.**
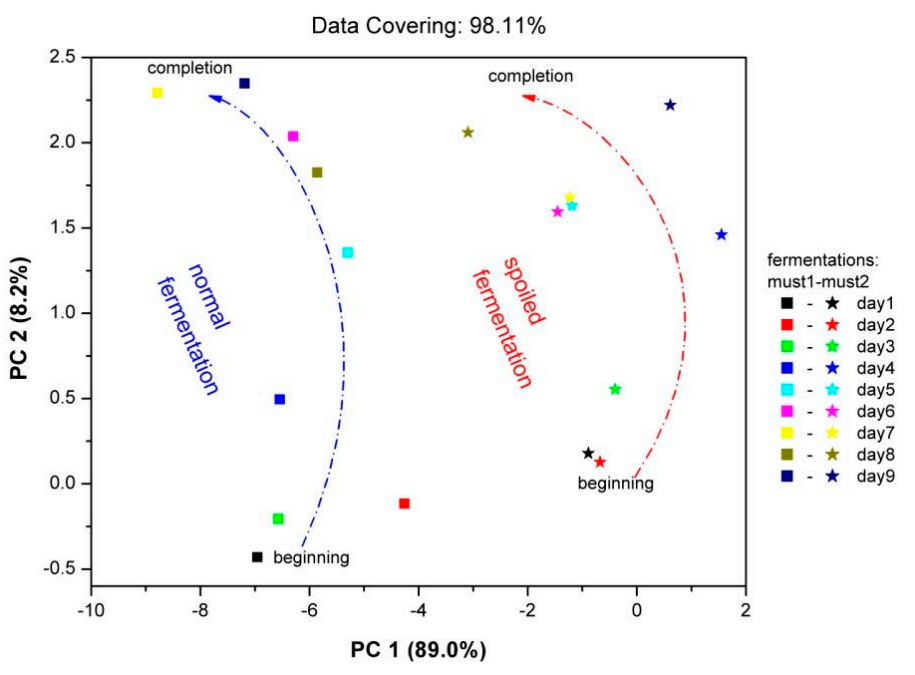
PCA analysis of the sensor array. Data processed: equilibrium responses during the fermentation progress.

With the use of the PCA data processing technique, mapping of the “fingerprint” of the sensor array to the complex vapor environment of fermenting must is attainable. Each fingerprint corresponds to a specific vapor environment. Therefore, in the same plot the fingerprints for both fermentations are imprinted (Figure 9). The results were autoscaled, in order to prevent high sensor responses from dominating the analysis and loose information from sensors with low responses respectively. On the Figure 9 axes, the percentage contribution of each principal component is shown. For both fermentations, the points corresponding to the beginning of fermentation process—situated at lower pc2 values—and to the final fermentation product—situated at higher pc2 values—are clearly discriminated. The early stages of two fermentations are clearly differentiated, in line with the corresponding different chemical analysis results. In each case, the path connecting the initial and final product is different related to the differentiation of two fermentation processes. The displacement of the fingerprints between two fermentation processes is related to the different chemical composition of both fermentations, since each point corresponds to different vapor environment. As an overview, it is obvious that the Must 2-”spoiled”-fermentation process is mapped in a different area of the PCA plot in comparison with the “fingerprints” of the Must 1-normal fermentation process.

## 4. Conclusions

A sensor array consisting of eight IDCs coated with different polymeric materials is used for the monitoring and evaluation of alcoholic must fermentations. The selection of the polymeric materials was based on previous studies on the sorption capacity of different polymeric materials to various VOCs and on the responses of polymer-based chemocapacitor arrays upon exposure to different VOCs and humidity [34,35]. Two musts with distinctly different fermenting characteristics, according to chemical analysis, were monitored by the sensor array.

The results obtained demonstrate that, without sample pretreatment, the sensor array can be potentially used as electronic nose for monitoring and evaluation of the alcoholic fermentation process of a typical grape must variety. Elimination of the interference signal of humidity, which is present at elevated concentrations in fermenting must samples, is achieved by following the proposed experimental protocol (by using as a reference signal the one produced by the headspace of pure water). Therefore the sensor array responses correspond to the volatile organic compounds contributing to the headspace profile of the fermenting medium.

It is evident that the sensor array not only perceives the ethanol evolution, but each sensor's response is affected by the presence of other volatile compounds produced during the fermentation process. The magnitude of the effect depends on: (a) the sensing polymeric material of the sensor and its interaction with the sorbed analytes, (b) the dielectric constant of sorbed analytes and (c) the volatility of each component which, in turn, is affected by the chemical affinity towards the other constituents of the multicomponent medium. By mapping the “fingerprints” of the sensor array with PCA, discrimination between a normal fermentation and a “spoiled” fermentation is performed. Particularly, for the examined “spoiled” fermentation, the PCA score is correlated with the undesirable enhancement of acetic acid and ethyl acetate. Both of these volatile compounds are related with spoiled organonoleptic properties of the final product. Even though this PCA pattern is not a quantitatively analytical tool of the fermenting medium composition, it can be used for control and evaluation of the fermentation procedure.

## Figures and Tables

**Table 1.** Concentrations of characteristic components of the fermented musts at the beginning and at the end of the fermentation processes.

**Fermentation Processes**	**Volatile Esters (Ethylacetate (ppm))**		**Total Acidity (Tartaric Acid (g/L))**	
	
	**beginning**	**end**	**beginning**	**end**
**Normal**	4	65	7.125	7.2
**Spoiled**	15	300	7.5	10.125

**Table 2.** Equilibrium Responses of two representative polymer coated sensors upon exposure to the headspace of several standard pure or contaminated with controlled concentrations of ethyl acetate and acetic acid in ethanol solutions.

	**Equilibrium Capacitance Responses dC (pF) [Mean Values and Standard Deviation (±)]**			

	**12% EtOH**	**2 g/L AcOH**	**12% EtOH + 2 g/L AcOH**	**12% EtOH + 2 g/L AcOH + 300 ppm EtOAc**
**PBMA**	0.0183 (±0.0019)	−0.0026 (±0.0004)	0.0179 (±0.0022)	0.0196 (±0.0012)
**PEI**	−0.0321 (±0.0011)	−0.0230 (±0.0050)	−0.0573 (±0.0033)	−0.0319 (±0.0058)
